# Exogenous leucine alleviates heat stress and improves saponin synthesis in *Panax notoginseng* by improving antioxidant capacity and maintaining metabolic homeostasis

**DOI:** 10.3389/fpls.2023.1175878

**Published:** 2023-04-19

**Authors:** Haijiao Liu, Yingwei Su, Yunxia Fan, Denghong Zuo, Jie Xu, Yixiang Liu, Xinyue Mei, Huichuan Huang, Min Yang, Shusheng Zhu

**Affiliations:** ^1^ State Key Laboratory for Conservation and Utilization of Bio-Resources in Yunnan, Yunnan Agricultural University, Kunming, China; ^2^ Key Laboratory for Agro-Biodiversity and Pest Control of Ministry of Education, Yunnan Agricultural University, Kunming, China

**Keywords:** leucine, *Panax notoginseng*, heat stress, antioxidant, carbohydrate metabolism

## Abstract

*Panax notoginseng* saponins (PNSs) are used as industrial raw materials to produce many drugs to treat cardio-cerebrovascular diseases. However, it is a heat-sensitive plant, and its large-scale artificial cultivation is impeded by high temperature stress, leading to decreases in productivity and PNSs yield. Here, we examined exogenous foliar leucine to alleviate heat stress and explored the underlying mechanism using metabolomics. The results indicated that 3 and 5 mM exogenous foliar leucine significantly alleviated heat stress in one-year- and two-year-old *P. notoginseng* in pots and field trials. Exogenous foliar leucine enhanced the antioxidant capacity by increasing the activities of antioxidant enzymes (POD, SOD) and the contents of antioxidant metabolites (amino acids). Moreover, exogenous foliar leucine enhanced carbohydrate metabolism, including sugars (sucrose, maltose) and TCA cycle metabolites (citric acid, aconitic acid, succinic acid and fumaric acid), in *P. notoginseng* leaves, stems, and fibrous roots to improve the energy supply of plants and further alleviate heat stress. Field experiments further verified that exogenous foliar leucine increased the productivity and PNSs accumulation in *P. notoginseng*. These results suggest that leucine application is beneficial for improving the growth and quality of *P. notoginseng* under heat stress. It is therefore possible to develop plant growth regulators based on leucine to improve the heat resistance of *P. notoginseng* and other crops.

## 1 Introduction

*Panax notoginseng* is one of the most important semi-shade industrial herbal medicines cultivated more than four hundred years in Southwest China (Yang et al., 2018; Ye et al., 2019). *P. notoginseng* saponins (PNSs) are the common active substances of *P. notoginseng*, including mainly ginsenosides Rg_1_, Rb_1_, Re, Rd and notoginsenoside R_1_ (Xia et al., 2017). Many preparations based on PNSs have been widely used in the prevention and treatment of cardio-cerebrovascular diseases, hypertension, atherosclerosis, etc. (Ye et al., 2019). By 2016, the planting area of *P. notoginseng* reached approximately 30,000 hectares, and the total output was approximately 45,000 kg (Cui and Huang, 2018). Currently, there are about 500 patented medicines that use the ingredients of *P. notoginseng*, and the market scale has reached approximately $1.5 billion (Cui and Huang, 2018; Xu et al., 2019). *P. notoginseng* grows healthily at 18~25°C, but high temperature above 30°C can cause serious thermal damage (Quan et al., 2018). Its cultivation risk is increasingly affected by abnormal warming. In particular, high temperature stress results in reduced productivity and poor quality of half-shadow herbal medicines (Lee et al., 2010; Kim et al., 2019). With global warming, high temperature stress has become an increasingly significant abiotic factor that affects plant growth and survival (Zhao et al., 2020; Shang et al., 2022). Elevated temperatures damage the plant cell membrane, causing leaf and stem wilting, scorching and abscission (Wang et al., 2019). Heat stress resulted in a decrease in the content of carbohydrate metabolites, and plants allocate more energy for stress response under elevated heat conditions (Wang et al., 2018). Large amounts of reactive oxygen species (ROS) accumulate due to heat stress, leading to cellular oxidative damage (Liu et al., 2021c). The increase in ROS levels will also slow down the flow of enzymes sensitive to inactivation by oxidation to the TCA cycle, such as aconitase and pyruvate dehydrogenase, and consequently weaken the energy supply of the cell under heat stress (Zhu, 2016; Choudhury et al., 2017), which eventually affects plant growth.

Plants cope with heat stress by ROS scavenging, renaturation or elimination of the denatured proteins and other regulatory mechanisms (Zhu, 2016). However, these processes are often accompanied by the undesirable side effect of reduced growth (Zhang et al., 2022). It is also of great significance in terms of genetically improving crops to identify heat resistance genes and study their regulatory mechanisms by genetic means (Kan et al., 2022). However, it is especially difficult to genetically manipulate certain economic and medicinal crops with low improvement and long growth cycles (Xu et al., 2021).

Many studies have indicated the functions of exogenous chemical compounds for plant priming to enhance heat stress tolerance, such as antioxidants (ascorbic acid, AsA), protectants (silicone, Si), and hormones (salicylic acid, SA) (Alayafi, 2020; Khan et al., 2020; Wassie et al., 2020). In recent years, some reports have noted that the exogenous application of amino acids can protect plants from abiotic stresses, such as glutamate (Li et al., 2019), arginine (Collado-González et al., 2020), and γ-aminobutyric acid (Kumar et al., 2019). Some amino acid, such as proline, can act as ROS scavenger to improve plant stress tolerance (Kaushik and Aryadeep, 2014). Branched-chain amino acids (BCAAs), including leucine, isoleucine and valine, can regulate plant stress tolerance through the plant respiratory system (Pires et al., 2016). More importantly, BCAAs play critical roles in the regulation of plant energy homeostasis (Mero, 1999; Nie et al., 2018). The carbon skeletons of BCAAs can convert to precursors or intermediates of TCA cycle for ATP production, and the energy yield of leucine is higher than that of isoleucine and valine (Hildebrandt et al., 2015). It was reported that the appropriate level of ROS can be used as a signal transduction molecule to regulate metabolism during abiotic stress, as long as the cells maintain enough high energy reserves to detoxify ROS (Choudhury et al., 2017). Therefore, it is worth exploring whether exogenous leucine maintains normal energy metabolism and thus enhances plant heat stress resistance.

In this study, we used *P. notoginseng* as a model medicinal plant to: (1) test whether exogenous foliar leucine could alleviate heat stress; (2) explore how exogenous foliar leucine enhances heat tolerance through metabolic changes; and (3) verify whether exogenous leucine spraying could relieve high temperature stress and improve productivity and the contents of PNSs in the field. Based on the above results, we hope to find a new strategy to alleviate heat stress for sustainable agricultural development under global warming conditions.

## 2 Materials and methods

### 2.1 Plant material and experimental design in the laboratory

One-year-old *P. notoginseng* seedlings grown in pots (top diameter:15 cm, height: 12.5 cm) in a semi-shaded greenhouse located in Xundian County, Yunnan Province, China (1,950 m, 25.5°N, 103.3°E) were selected as the materials for the laboratory experiments. The experiments were conducted as follows. First, healthy, uniform *P. notoginseng* plants were transported to the laboratory and kept in incubators at 20°C (light, 9 h; dark, 15 h) to rejuvenate growth for 24 h. After that, the seedlings were heated to 36°C (light period, 9 h)/20°C (dark period, 15 h) for 15 days in the incubators. During this time, *P. notoginseng* leaves were evenly sprayed with different concentrations of leucine (1, 3 and 5 mM) once every two days. Sterile deionized water was used as the control treatment. Each treatment group consisted of 6 pots. Each plot was sprayed with 3 mL of leucine solution or sterile deionized water. The soil water content was maintained at 25%-27% by replenishing the soil water the day after each leucine spraying treatment. The positions of the pots in the incubator were changed once a day to prevent uneven temperature and light conditions. After 15 days, the number of damaged leaves (those exhibiting symptoms such as drying, yellowing, wilting and dropping) of *P. notoginseng* was counted, and the rate of damaged leaves was calculated according to the following formula:

Rate of damaged leaves (%) = number of damaged leaves/total number of leaves of the plant × 100.

Next, the *P. notoginseng* plants, including leaves, stems and fibrous roots, were separated and stored at -80°C after rapid freezing with liquid nitrogen for subsequent determinations.

### 2.2 Determination of antioxidant enzyme activity

The SOD (superoxide dismutase) activity of the *P. notoginseng* leaves was quantified using a WST-8 assay kit (Suzhou Keming Biotechnology Co., Ltd.) following previous reports (Cao et al., 2022). In brief, 0.1 g of leaves and 1 mL of extraction solution are ground into homogenate on ice, and then centrifuged at 8000 g and 4°C for 10 min, taking the supernatant as the extract. After treatment, the extract was incubated with reaction solution for 30 min at 25 °C. The absorbance at a wavelength of 450 nm was measured with an enzyme-labeled instrument (Molecular Devices, Sunnyvale, CA, United States). Another assay kit (Nanjing Jiancheng Bioengineering Institute, Nanjing, China) was used to measure the POD (peroxidase) enzyme activity of *P. notoginseng* leaves (Hu et al., 2022). Briefly, 0.1 g of leaves and 0.9 mL physiological saline solution were homogenized and centrifuged for 10 min at 4°C and 8000 g. The enzyme activity was measured by taking the supernatants and determining its absorbance at the wavelength of 470 nm on enzyme-labeled instrument.

### 2.3 Metabolite extraction and analysis

The changes in metabolites of *P. notoginseng* was measured following previous report with some modifications (Ji et al., 2017). The plant tissues of *P. notoginseng* ground in liquid nitrogen, including leaves, stems and fibrous roots, were used for metabolite extraction. Leaves treated with 1, 3, and 5 mM leucine and sterile deionized water were labelled L_1, L_3, L_5, and L_CK, respectively. Similarly, the stems and fibrous roots were labelled S_1, S_3, S_5, and S_CK and R_1, R_3, R_5, and R_CK, respectively. Approximately 60 mg samples of *P. notoginseng* tissues were weighed and ultrasonically extracted at 37°C for 30 min with 1 mL of extraction solution prepared by methanol, chloroform and sterilized deionized water in a volume ratio of 5:2:2. Then the homogenates were centrifuged for 3 min (1600 g, 4°C). After that, the supernatant was transferred and dried using a SpeedVac (Christ, Germany) at 25°C. A total of 80 μL of 20 mg·mL^−1^ methoxyamine hydrochloride solution dissolved in pyridine and 40 μL of N-methyl-N-(trimethylsilyl)-trifluoroacetamide were added to the dried samples in two steps, and reacted for 90 min, 30°C and 30 min, 37°C respectively for derivatization. Finally, each sample was centrifuged for 3 min (1600 g, 4°C) and stored at 4°C for metabolite analysis.

Based on a previously reported method (Liu et al., 2021b), gas chromatography−mass spectrometry (GC−MS; QP2010 Ultra, Shimadzu, Kyoto, Japan) was used to analyse the metabolites of *P. notoginseng* tissues. The model of chromatographic column was SH-Rxi-5Sil MS and 30.0 m × 0.25 mm × 0.25 µm. The offline data was first converted to abf format using Analysis Base File Converter, and then processed by MSDIAL for peak search, peak alignment, and identification of metabolite ion peaks. Principal component analysis (PCA) was conducted by Majorbio (https://cloud.majorbio.com/), and orthogonal projection to latent structures-discriminant analysis (OPLS-DA) was conducted by MetaboAnalyst 4.0 (http://www.metaboanalyst.ca/MetaboAnalyst/). Based on a fold change (FC) >2 or <0.5 and *p* < 0.05, the differentially accumulated metabolites (DAMs) were screened and then mapped to biological pathways in the Kyoto Encyclopedia of Genes and Genomes (KEGG) database. Correlation analysis between the DAMs and morphological and physiological responses was conducted using OmicStudio (https://www.omicstudio.cn/).

### 2.4 Plant material and verification experiment in the field

Field experiments were conducted to verify the effects of exogenous leucine on the heat stress tolerance, biomass and quality of *P. notoginseng* in Lancang County, Yunnan, China (99.48°E, 22.49°N; altitude of 1,560 m). The experimental sites were on sunny slopes where the daily maximum temperature of the two planting bases could reach 35°C for approximately 2 h from 14:00 to 16:00. The average daily temperature was approximately 25°C. One-year-old *P. notoginseng* seedlings grown in a semi-shaded greenhouse and two-year-old *P. notoginseng* plants grown under *Pinus kesiya* var. *langbianensis* forest were selected as experimental materials. The field plot area was 1.0 × 0.5 m, and six replicate samples were randomly selected. Approximately 100 seedlings were retained in each treatment plot of one-year-old *P. notoginseng*, and 20 plants were retained in each treatment plot of two-year-old *P. notoginseng*. The concentrations of leucine sprayed on the leaves of the one-year- and two-year-old *P. notoginseng* were the same as those in the laboratory, but the field experiments were conducted for a longer period of 30 days. Leucine was sprayed every three days, and other management measures were the same as those in the field.

After 30 days of heat stress, the one-year-old *P. notoginseng* leaves mainly showed yellow spot symptoms; hence, the number of yellow-spotted leaves was counted and calculated according to the following formula:

Rate of yellow-spotted leaves (%) = number of yellow-spotted leaves/total number of leaves of the plant × 100.

The two-year-old *P. notoginseng* leaves exhibited symptoms of drying and yellowing in the leaf margin, so the rate of damaged leaves was calculated with the same formula as that used for the laboratory experiments. In addition, all plants were harvested and dried in an oven at 60°C to measure the dry weights. Furthermore, the most important medicinal part, the dried rhizomes and roots, were beaten into fine powder and stored at 4°C for determination of PNSs content.

### 2.5 PNSs extraction and content analysis

The PNSs contents in the rhizomes and roots of one-year- and two-year-old *P. notoginseng* were measured by ultra-performance liquid chromatography (UPLC) following the reported method (Yang et al., 2015). Briefly, 0.2 g of *P. notoginseng* rhizomes and roots fine powder and 15 mL of 70% methanol solution were ultrasonicated for 30 min, and then the suspension was centrifuged for 5 min at 4°C, 12,000 g. The supernatants were filtered through a 0.22 µm nylon membrane filter and quantified using a Nexera X2 UPLC system (Shimadzu, Japan) equipped with a diode array detector (DAD) and a Poroshell 120 EC-C_18_ reversed-phase column (150 × 4.6 mm, 4 µm, Agilent). The UPLC parameters were described in detail in our previous report (Liu et al., 2021a).

### 2.6 Statistical analysis

SPSS 26.0 was used for statistical analyses with one-way analysis of variance (ANOVA) and Duncan’s multiple range test (*p* < 0.05). GraphPad Prism 8.0 was used for analysing the raw data and graph construction. Spearman correlation coefficient was employed to correlate the DAMs with the morphological and physiological indicators.

## 3 Results

### 3.1 Exogenous spraying of leucine onto leaves alleviates heat stress in the laboratory

**Figure 1:**
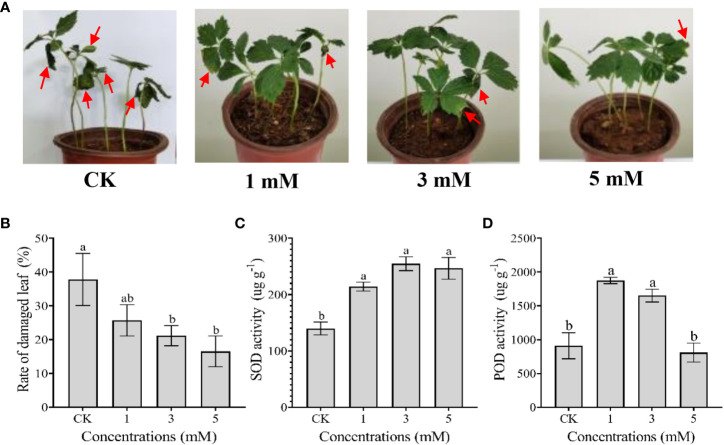
Effect of spraying leaves with exogenous leucine on the morphological and physiological responses of *P. notoginseng* under heat stress in the laboratory. **(A)** Morphology of *P. notoginseng* seedlings treated with sterilized deionized water (CK) and exogenous leucine at concentrations of 1, 3 and 5 mM once every two days while under heat stress for 15 days. **(B)** Changes in the damaged leaf rate of *P. notoginseng* treated with CK and leucine after 15 days of heat stress. **(C, D)** Changes in the activities of SOD and POD in *P. notoginseng* treated with CK and leucine after 15 days of heat stress, respectively. Error bars indicate the standard error (SE) calculated from six biological replicates. Different letters indicate statistically significant differences among the treatments by Duncan’s multiple range test at *p* < 0.05.

After exposure to 36°C heat stress for 15 days, the leaves of *P. notoginseng* showed thermal damage, as evidenced by leaf burning, withering and curling (**Figure 1A**). Exogenous leucine spraying alleviated the symptoms of thermal damage, and the effects were dose-dependent (**Figure 1A**). The rates of damaged leaves treated with 3 and 5 mM leucine were significantly lower than that of the control group (**Figure 1B**). Moreover, exogenous leucine spraying significantly increased the activities of the antioxidant enzymes SOD (1, 3 and 5 mM) and POD (1 and 3 mM) (**Figures 1C, D**).

### 3.2 Exogenous leucine changes the metabolic profile of *Panax notoginseng*

**Figure 2:**
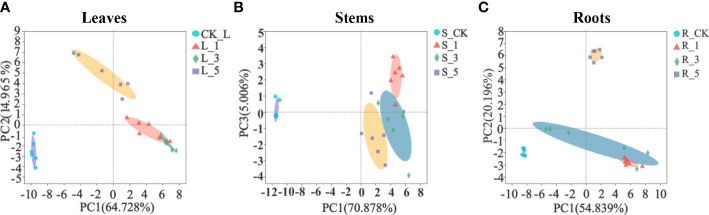
Principal component analysis (PCA) of the metabolites in *P. notoginseng*. **(A–C)** Represent the leaves, stems and fibrous roots, respectively.

A total of 67 metabolites in the control and leucine treatments were identified by GC−MS, including 17 sugars and derivatives, 16 organic acids, 19 amino acids and derivatives, 4 polyamines, 2 alcohols and 9 other metabolites. PCA was used to distinguish the differences in the metabolites in the leaves, stems and fibrous roots of *P. notoginseng*. The two principal components (PC1 and PC2 or PC3) of the leaves, stems and fibrous roots accounted for 64.728% and 14.965%, 70.878% and 5.006%, 54.839% and 20.196% of the variance, respectively. These three tissues could separate the leucine-treated leaves from the corresponding control (**Figures 2A–C**), which indicated that spraying leaves with leucine strongly affected the whole plant metabolite profile under high temperature stress. In addition, treatment with 1 and 3 mM leucine also tended to separate from 5 mM treatment in the leaves (**Figure 2A**) and fibrous roots (**Figure 2C**).

**Figure 3:**
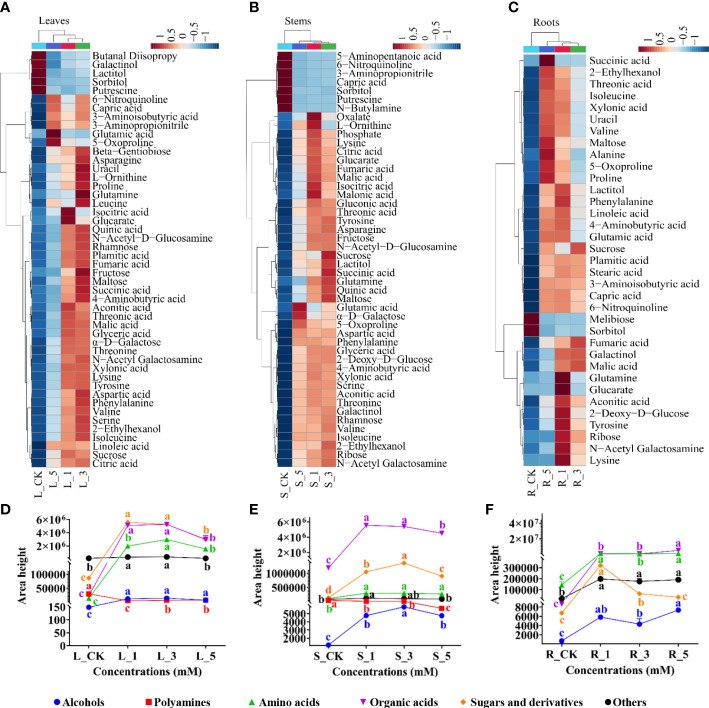
Changes in the abundances of the common DAMs in leaves, stems and fibrous roots. **(A–C)** Represent the leaves, stems and fibrous roots, respectively. **(D–F)** Represent the relative abundances of the different classes of common DAMs in the leaves, stems and fibrous roots, respectively. Error bars indicate the standard error (SE) calculated from six biological replicates. Different letters indicate statistically significant differences among the treatments by Duncan’s multiple range test at *p* < 0.05.

OPLS-DA was further used to screen the DAMs among the nine groups (**Supplementary Figure S1**; **Supplementary Table S1**) and a total of 52, 55, 52, 55, 53, 49, 57, 64, 60 DAMs were found the in paired groups L_CK/L_1, L_CK/L_3, L_CK/L_5, S_CK/S_1, S_CK/S_3, S_CK/S_5, R_CK/R_1, R_CK/R_3, R_CK/R_5 respectively (**Supplementary Figure S2A**). A Venn diagram further revealed 48, 48 and 35 common DAMs in the leaves, stems and fibrous roots that were regulated by different concentrations of leucine (**Figures S2B–D**). A cluster heatmap was generated to exhibit the changes in the relative contents of the common DAMs among the leucine concentrations (1, 3 and 5 mM) and their corresponding control group. The DAMs in the three tissues of *P. notoginseng* after treatment with 1, 3 and 5 mM leucine clustered into one branch with the control group constituting another branch. Most of the DAMs had higher contents in the leucine-treated groups (**Figures 3A–C**).

DAMs were classified as alcohols, polyamines, amino acids, organic acids, sugars and derivatives, and others. Interestingly, all classes of DAMs in the leaves and stems were significantly increased after 1 and 3 mM leucine treatment with the exception of polyamines, which were in the highest abundance in the control group (**Figures 3D, E**). For the fibrous roots, sugars and derivatives and amino acids had significantly accumulated in the 1 mM leucine treatment group. Alcohols and organic acids had the highest abundance after 5 mM leucine treatment (**Figure 3F**).

### 3.3 Exogenous leucine enhances amino acid and carbohydrate metabolism

KEGG enrichment was carried out to better understand the biological functions of the DAMs in the leaves, stems and fibrous roots of *P. notoginseng* under heat stress. The results indicated that 44, 43 and 40 pathways were enriched by the DAMs in the leaves, stems and fibrous roots, respectively (**Supplementary Figure S3**; **Supplementary Table S2**). There were 19, 17 and 16 pathways with an impact >0 in the leaves, stems and fibrous roots, which were mainly classified as amino acid metabolism (8, 8 and 7) and carbohydrate metabolism (6, 6 and 5) (**Supplementary Figures S4**–**S6**).

**Figure 4:**
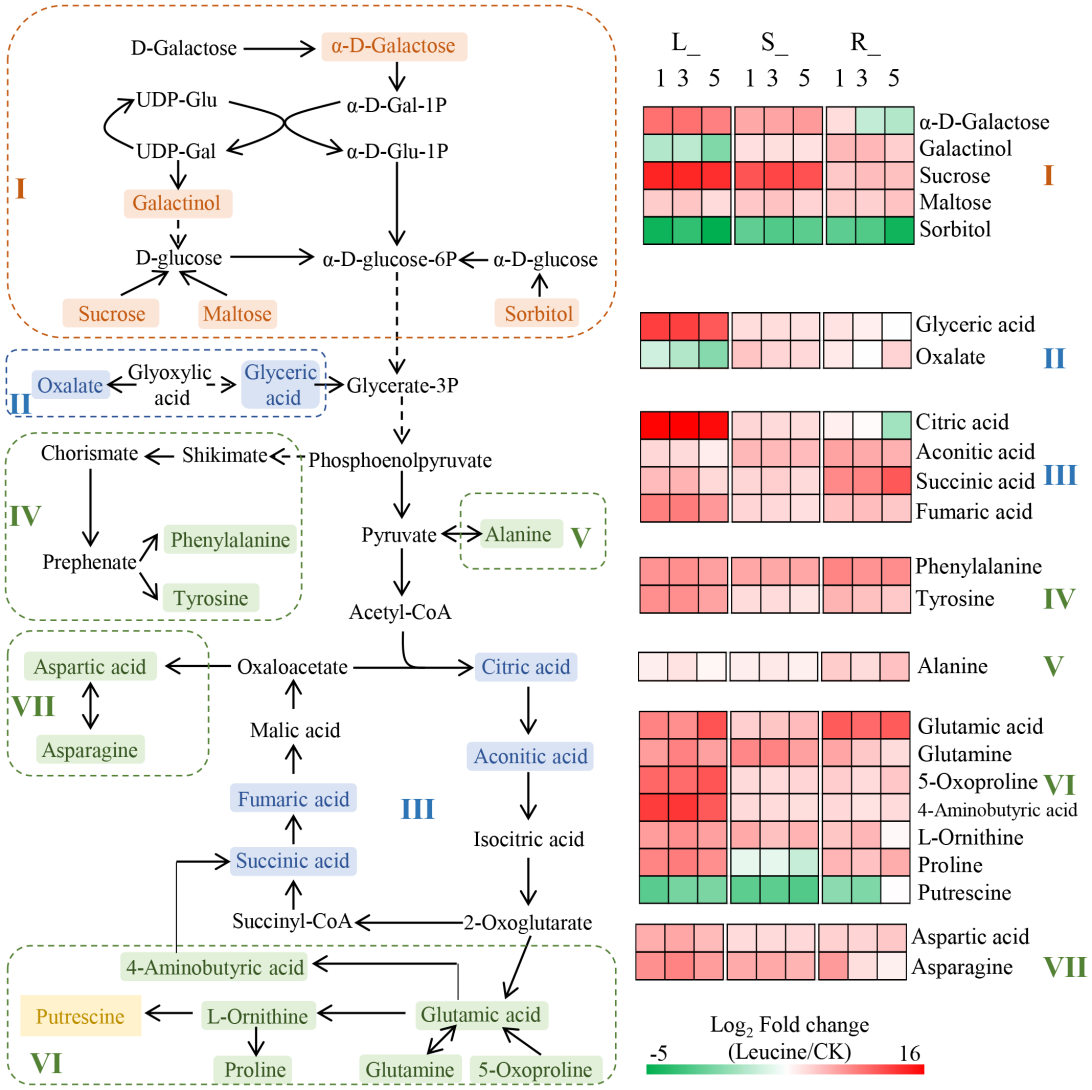
KEGG metabolic network analysis of the pathways in the leaves, stems and roots. Parts I, II and III are the decomposition of disaccharides or monosaccharides in glycolysis and then influx into the TCA cycle. Parts IV, V, VI and VII are amino acid biosynthesis metabolism pathways using the intermediate products of glycolysis and the TCA cycle as precursors. DAMs shown in orange, blue, green and yellow represent sugars and derivatives, organic acids, amino acids and polyamines, respectively. The colour block represents the fold changes (leucine/CK) after log_2_ conversion; red represents upregulation, and green represents downregulation.

To comprehensively understand how leucine spraying effects the metabolism of *P. notoginseng* seedlings under heat stress, we constructed an overall metabolic pathway network based on the literature and the KEGG metabolic pathway database analysis. First, 14 shared metabolic pathways (pathway impact >0) in the leaves, stems, and fibrous roots were selected, including 7 involved in amino acid metabolism, 5 involved in carbohydrate metabolism, 1 involved in the biosynthesis of other secondary metabolites and 1 involved in energy metabolism (sulfur metabolism) (**Supplementary Figure S7**). Second, 23 DAMs in the leaves, stems and fibrous roots involved in the shared pathways were identified, including 5 sugars and derivatives, 6 organic acids, 11 amino acids and 1 polyamine. Finally, the integrated metabolic network was constructed according to the association between the metabolic pathways recorded in the literature and the KEGG metabolic pathway database (**Figure 4**). The KEGG network was mainly composed of carbohydrate metabolism and related amino acid metabolism pathways. Parts I, II and III are the decomposition of disaccharides or monosaccharides in glycolysis and then influx into the TCA cycle, including the pathways of “galactose metabolism”, “starch and sucrose metabolism”, “glyoxylate and dicarboxylate metabolism”, “TCA cycle” and “butanoate metabolism”. Parts IV, V, VI and VII are amino acid biosynthesis metabolism pathways using the intermediate products of glycolysis and the TCA cycle as precursors, including “phenylalanine, tyrosine and tryptophan biosynthesis”, “phenylalanine metabolism”, “tyrosine metabolism”, “alanine, aspartate and glutamate metabolism”, “arginine biosynthesis”, “arginine and proline metabolism”, and “glutathione metabolism”. In addition, tyrosine is involved in “isoquinoline alkaloid biosynthesis”, and succinic acid is involved in “sulfur metabolism”.

Overall, exogenous leucine treatment enhanced both amino acid metabolism and carbohydrate metabolism in *P. notoginseng*. Specifically, the relative contents of most amino acids in the leaves, stems and fibrous roots after treatment with 1, 3 and 5 mM leucine were significantly increased under heat stress. The fold changes in tyrosine, 5-oxoproline, 4-aminobutyric acid, L-ornithine, proline, aspartic acid, and asparagine upregulation in the leaves were higher than those in stems and fibrous roots. Citric acid and fumaric acid, which are involved in the TCA cycle, also accumulated at high levels in the leaves. In addition, the contents of carbohydrates, including sucrose and α-D-galactose, were elevated to a greater extent in the leaves and stems than in the fibrous roots.

### 3.4 DAMs associated with morphological and physiological responses

**Figure 5:**
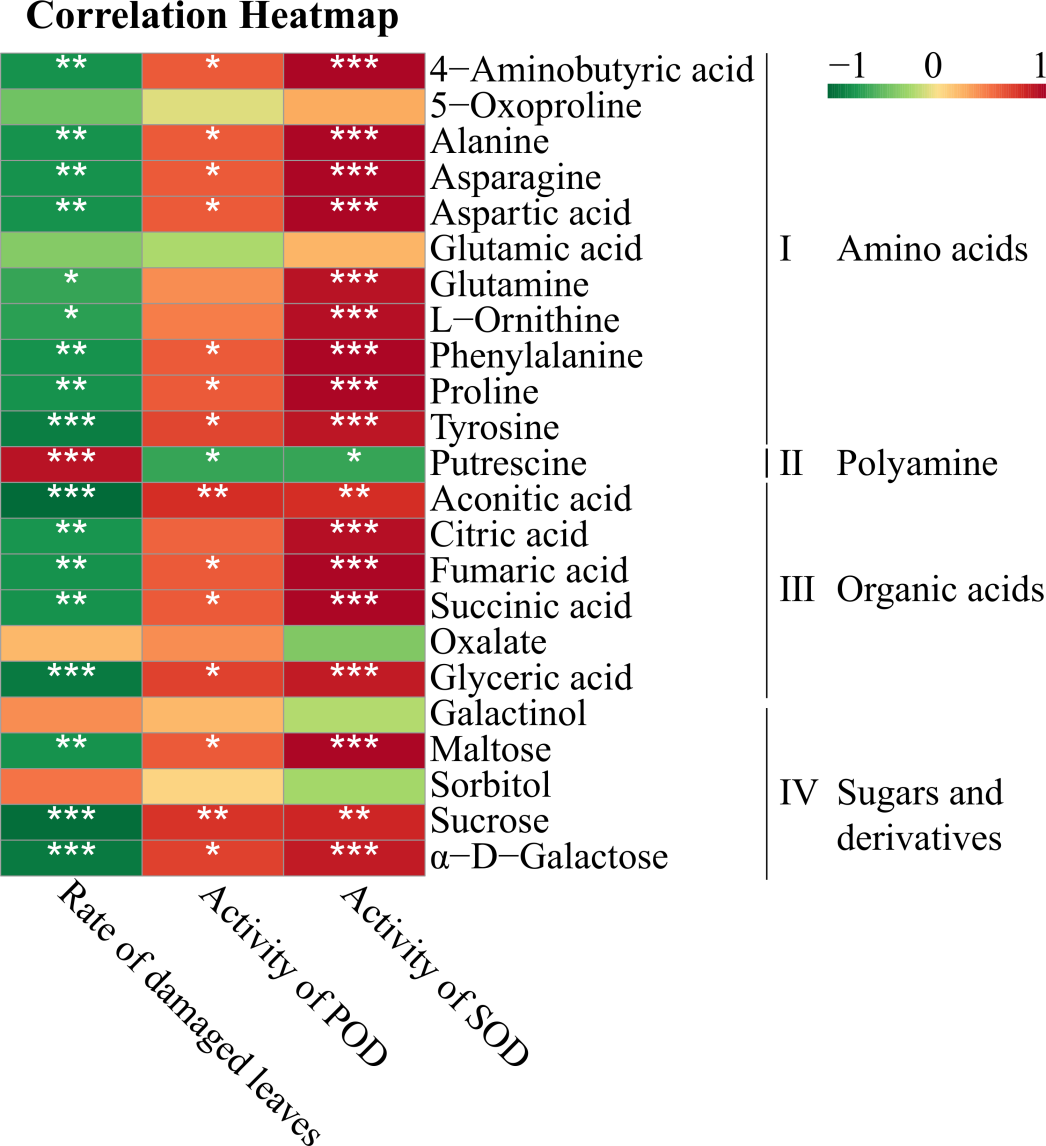
Spearman correlation analysis between the morphological and physiological responses and DAMs involved in the metabolic networks of the leaves. * Indicates a significant correlation at *p* < 0.05, ** indicates a significant correlation at *p* < 0.01 and *** indicates a significant correlation at *p* < 0.001. Red boxes represent positive correlations, green boxes represent negative correlations, and the depth of the colour represents the degree of the correlation.

Correlation analysis was conducted to determine the correlations between the morphological and physiological responses and DAMs in the leaves that are involved in the metabolic network (**Figure 5**). A total of 23 DAMs were classified into four groups: amino acids (part I), polyamines (part II), organic acids (part III) and sugars and derivatives (part IV). All amino acids, especially 4-aminobutyric acid, alanine, asparagine, aspartic acid, phenylalanine proline and tyrosine, were significantly negatively correlated with the rate of leaf disease and positively correlated with the activities of the antioxidant enzymes SOD and POD. In contrast, putrescine was negatively correlated with antioxidant enzymes and significantly positively correlated with the rate of damaged leaves. The correlations between the morphological and physiological indexes and citric acid, aconitic acid, succinic acid, fumaric acid and glyceric acid, which are involved in the TCA cycle or are precursors that influx into glycolysis, were consistent with those with the amino acids. Among sugars, only sucrose, maltose and α-D-galactose had significant negative correlations with the damaged leaf rate and significant positive correlations with antioxidant enzymes.

### 3.5 Exogenous leucine alleviates heat stress and increases biomass and PNSs content in the field

**Figure 6:**
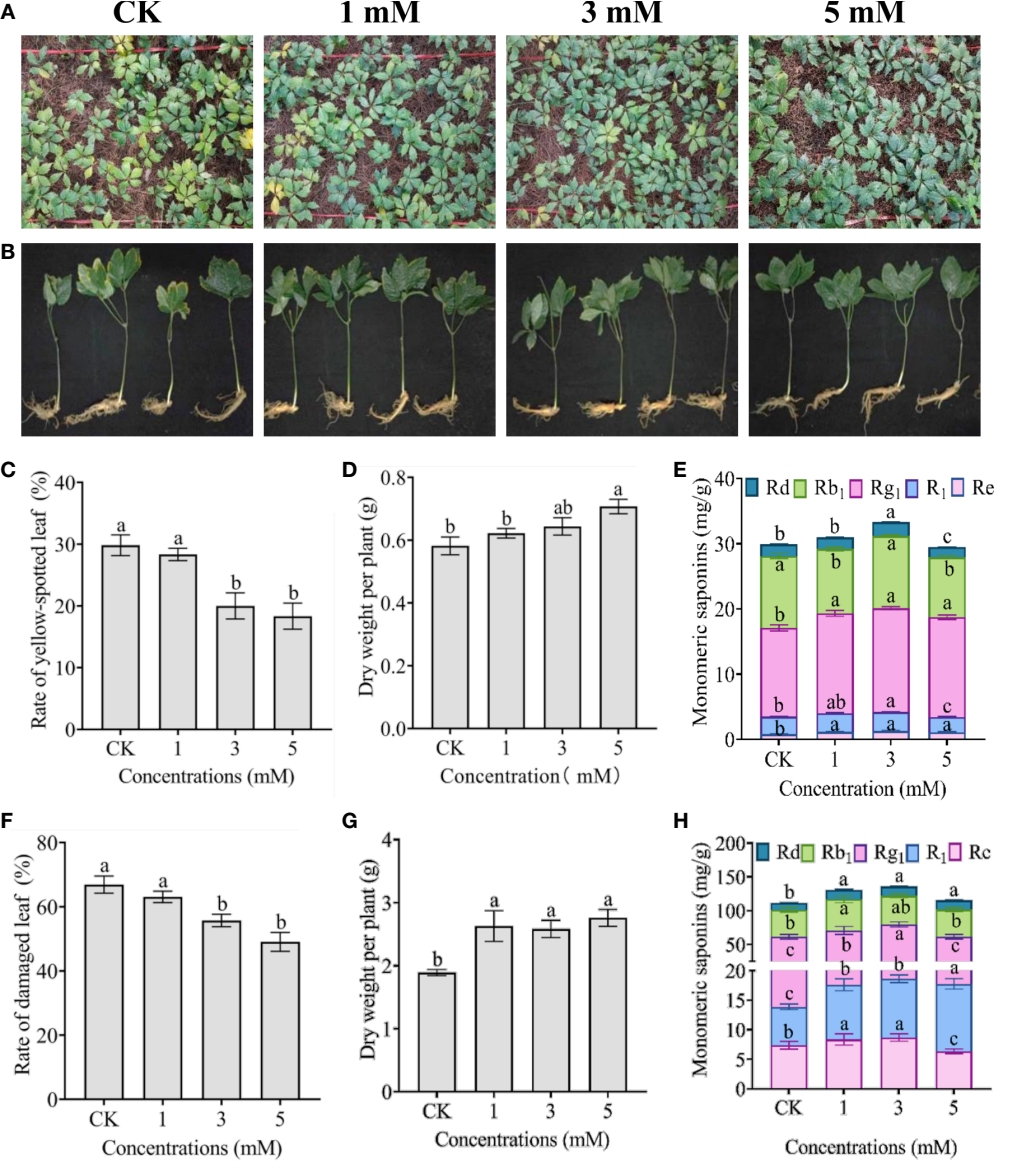
Effect of exogenous foliar leucine on the biomass and PNSs in *P. notoginseng* under heat stress in the field. **(A, B)** Morphological responses of one- and two-year-old *P. notoginseng* treated with sterilized deionized water (CK) and exogenous leucine at concentrations of 1, 3 and 5 mM once every three days under heat stress for 30 days in the field, respectively. **(C, F)** Changes in the damaged leaf rate of one- and two-year-old *P. notoginseng*, respectively. **(D, G)** Changes in the dry weight of one- and two-year-old *P. notoginseng* per plant, respectively. **(E, H)** Changes in the PNSs contents in one- and two-year-old *P. notoginseng* rhizomes and roots, respectively. Error bars indicate the standard error (SE) calculated from six biological replicates. Different letters indicate statistically significant differences among the treatments by Duncan’s multiple range test at *p* < 0.05.

Field experiments further verified that exogenous foliar leucine could alleviate heat stress in one- and two-year-old *P. notoginseng* (**Figure 6**). After 30 days of treatment at the maximum daily temperature of 35°C for approximately 2 h, approximately 30% of the one-year-old *P. notoginseng* leaves exhibited yellow spot symptoms, and 70% of the two-year-old *P. notoginseng* leaves exhibited marginal scorching (**Figures 6A, B**). However, the thermal damage was significantly alleviated by exogenous foliar leucine spraying at concentrations of 3 and 5 mM (**Figures 6C, F**). Exogenous foliar leucine increased the dry weight of *P. notoginseng* per plant in a dose-dependent manner (**Figure 6D**). For two-year-old *P. notoginseng*, the dry weight per plant increased significantly after treatment with 1, 3 and 5 mM exogenous leucine (**Figure 6G**). The dry weights of one- and two-year-old *P. notoginseng* treated with 5 mM leucine were 1.22 and 1.46 times higher than that of the control, respectively. More importantly, the total PNSs contents increased with certain leucine concentrations, especially 1 and 3 mM. The PNSs contents in one- and two-year-old *P. notoginseng* treated with 3 mM leucine were 1.11 and 1.22 times higher than that in the control, respectively (**Figures 6E, H**). In terms of monomer saponins, with an increase in leucine concentration, the contents of Rg_1_ and Re in one-year-old *P. notoginseng* first increased significantly and then decreased slightly, which was similar to the trend in two-year-old *P. notoginseng*. The application of 1, 3 and 5 mM leucine significantly increased the contents of Rd and R_1_ in two-year-old *P. notoginseng*; however, only treatment with 3 mM leucine produced a significant improvement in one-year-old *P. notoginseng*. Treatment with 1 mM leucine significantly increased the content of Rb_1_ in two-year-old *P. notoginseng* but had no effect on one-year-old *P. notoginseng* (**Figures 6E, H**).

## 4 Discussion

Heat stress caused by high temperature is one of the most important abiotic factors restricting crop yield (Zhang and Yang, 2019). In addition to genetic improvement to obtain crops with heat tolerance, the spraying of exogenous chemical compounds to prime plants for enhanced heat stress resistance is an alternative strategy, especially for some economic and medicinal crops with low improvement, long growth cycles and difficult genetic manipulation. *P. notoginseng*, as a very important raw medicinal herb, is threatened by heat stress, and it is difficult to improve its heat resistance by genetic manipulation. Here, experiments in the laboratory and in the field showed that exogenous leucine could effectively alleviate heat stress and promote productivity and PNSs accumulation. Metabolomic analysis combined with physiological tests validated that leucine could improve the antioxidant capacity and maintain metabolic homeostasis of *P. notoginseng* under heat stress. This study provides a new strategy to cope with heat stress using exogenous leucine spraying.

### 4.1 Exogenous leucine enhances the antioxidant capacity

Abiotic stresses, including heat, drought, cold, and salinity, will lead to ROS accumulation (Nadarajah, 2020). Heat stress stimulates ROS production because it increases the levels of the superoxide anion ( ${\text{O}}_{2}^{\;-}$ ), hydrogen peroxide (H_2_O_2_) and the hydroxyl radical (HO·) in the mitochondria while reducing the levels of SOD-1 mRNA, the cytoplasmic SOD protein and SOD enzymatic activity (Slimen et al., 2014). Excessive accumulation of these oxygen radicals results in cellular damage and death (Choudhury et al., 2017; Nadarajah, 2020). However, plants have evolved mechanisms to balance the production and scavenging of ROS through enzymatic and nonenzymatic antioxidative processes (Nadarajah, 2020). High antioxidant capacity, such as that obtained by enhancing the activities of SOD, POD, catalase (CAT), ascorbate peroxidase (APX), and glutathione reductase (GR), is conducive to improving the heat tolerance of plants (Wang et al., 2019; Tiwari et al., 2022). In this study, we found that spraying leucine at concentrations of 1 and 3 mM significantly increased the activities of the antioxidant enzymes SOD and POD, eventually decreasing the disease rate in the leaves of *P. notoginseng* under heat stress (**Figure 1**). Leucine application enhanced the antioxidant enzymes capacity SOD and POD of *P. notoginseng*, which is consistent with the results of GABA (Kumar et al., 2019), proline (Ben Ahmed et al., 2010), citrulline (Ahmadi et al., 2020), etc. enhancing the antioxidant enzyme capacity of other plants. The underlying mechanism deserves further study.

In addition, nonenzymatic antioxidants play an important role in the improvement in plant antioxidant capacity (Nadarajah, 2020). In this study, metabolomics analysis showed that the contents of 11 amino acids (phenylalanine, tyrosine, alanine, glutamic acid, 5-oxoproline, glutamine, L-ornithine, proline, 4-aminobutyric acid, aspartic acid, and asparagine) synthesized from four different precursors (phosphoenolpyruvate, pyruvate, 2-oxoglutarate and oxaloacetate) significantly increased after leucine application (**Figure 4**). As previously reported, the exogenous application of some amino acids can enhance the antioxidant capacity of plants in different ways (Haghighi et al., 2022). For example, aromatic amino acids (phenylalanine, tyrosine, etc.) can contribute their own electrons to convert oxygen radicals into stable substances (Deng et al., 2022). Exogenous proline and 4-aminobutyric acid reduce the oxidative damage of barley seedlings under aluminium and proton stress by enhancing the activities of the antioxidant enzymes SOD, POD and CAT (Song et al., 2010; Guo et al., 2022). Acidic amino acids, including glutamic acid and aspartic acid, can act as proton donors to pair with lone pair electrons and thus exert antioxidant effects (Zhang et al., 2016; Li et al., 2021) or enhance the antioxidant capacity by increasing antioxidant enzyme activity (Liu and Yang, 2015; Fardus et al., 2021). Moreover, glutathione is the most important nonenzymatic antioxidant in plants, which can by synthesized from glutamic acid, which itself can be synthesized from 5-oxoproline in the glutathione synthesis pathway (Xu et al., 2013; Liu et al., 2021b). Glutamine and asparagine have important physiological regulatory functions and play protective roles in antioxidant processes by increasing the vital antioxidant biomolecules/enzymes (Wang et al., 2004; Kaya et al., 2022) or being converted into glutamic acid and aspartic acid by deamination to exert antioxidant effects (Li et al., 2010). The effects of alanine and L-ornithine on the antioxidant capacity of plants has not been clearly demonstrated.

Also of note is that the content of putrescine, a polyamine metabolite, decreased significantly after the application of leucine (**Figure 4**). Previous studies have reported that putrescine is a key metabolite involved in plant development, tolerance and resistance to stress (Groppa and Benavides, 2008; González-Hernández et al., 2022). However, excessive putrescine can also produce toxic effects on plants (Capell et al., 1998). Therefore, plants have evolved an ingenious strategy to regulate the expression of the S-adenosylmethionine decarboxylase gene *SAMDC* to increase the content of putrescine under stress and reduce it after stress is resolved (Takahashi and Kakehi, 2010; Liu et al., 2011). In this study, the decrease in putrescine content may be related to the relief of *P. notoginseng* heat stress provided by leucine.

In summary, foliar spraying of leucine enhanced the activity of antioxidant enzymes and increased the contents of nonenzymatic antioxidant metabolites (amino acids), which improved the antioxidant capacity of *P. notoginseng* under heat stress and thus reduced the rate of damaged leaves (**Figures 1B**, **6C, F**). These data were consistent with the results of the correlation analysis showing that all 11 amino acids were negatively correlated with the damaged leaf rate (**Figure 5**). In the field, the productivity of one- and two-year-old *P. notoginseng* increased after leucine application (**Figures 6D, G**). The increase in biomass may be related to the effect leucine application had on reducing the rate of *P. notoginseng* damaged leaves, enabling them to perform adequate photosynthesis and then resume normal growth.

### 4.2 Leucine maintains metabolic homeostasis by regulating carbohydrate metabolism

High temperatures that cause heat stress disturb cellular metabolic homeostasis and impede growth and development in plants (Hasanuzzaman et al., 2013; Li and Howell, 2021). Studies have confirmed that heat stress can interfere with normal carbohydrate metabolism in plants (Shan et al., 2021). For example, heat stress reduced the accumulation of total soluble sugars, starch and carbohydrate metabolism enzymes in wheat (Iqbal et al., 2021). Under 35°C heat stress, the contents of citric acid and 2-oxoglutarate in *Isochrysis galbana* were reduced, indicating that the TCA cycle, the most important energy supply pathway in plants, was severely damaged (Su et al., 2017). In this study, foliar spraying of exogenous leucine altered the metabolic profile of *P. notoginseng* (**Figure 2**), and especially enhanced carbohydrate metabolism (**Figure 4**). Some soluble sugars (sucrose, maltose, α-D-galactose and galactinol) and organic acids (citric acid, aconitic acid, succinic acid, fumaric acid, glyceric acid, oxalate) involved in carbohydrate metabolism increased significantly in the leaves, stems and fibrous roots (**Figure 4**).

Soluble sugars are closely associated with plant growth and heat stress tolerance because they are directly involved in the synthesis of other compounds (Rajam et al., 1998; Xu et al., 2013). Plants will adjust their sucrose metabolism strategy and release sugars and other derived metabolites to support their growth under stress, as these compounds act as osmotic protectants to mitigate the negative effects of stress (Rizhsky et al., 2004). *In vitro* assays demonstrated that maltose can protect proteins, membranes, and the photosynthetic electron transport chain at physiologically relevant concentrations under abiotic stress (Kaplan and Guy, 2004). In addition, sucrose and maltose are important energy carriers and can be hydrolysed into glucose for energy production (Krasensky and Jonak, 2012; Yue et al., 2021). According to the KEGG metabolic pathway analysis, galactinol and α-D-galactose can also be directly involved with or converted into D-glucose to participate in glycolysis and the TCA cycle. Moreover, as a compatible solute, an increase in galactinol content is beneficial to membrane protection and radical scavenging and thus enhances the stress resistance of plants (Song et al., 2016; Liu et al., 2021d). The content of sorbitol was significantly reduced after leucine application (**Figure 4**). The increase in sorbitol synthesis in tomato leaves may result in improved salt stress tolerance (Tari et al., 2010). Whether sorbitol alleviates heat stress in *P. notoginseng* needs to be further confirmed, and how leucine changes the content of sorbitol also needs to be further explored.

Heat stress can reduce the contents of organic acids and the activities of key enzymes involved in the TCA cycle, inhibit the most critical energy metabolic process that drives ATP synthesis and energy supply, and ultimately affect the growth of plants (Zhu, 2016; Choudhury et al., 2017; Su et al., 2017). This study found that spraying exogenous leucine on the leaves of *P. notoginseng* significantly increased the contents of four important organic acids involved in the TCA cycle when compared with the control treatment under heat stress (**Figure 4**). In addition, the content of glyceric acid also increased. This may be conducive to increasing the content of glycerate-3P, which is an important intermediate metabolite in glycolysis and the TCA cycle. Combined with the change in soluble sugar content, we speculate that sucrose and maltose, as energy carriers, are hydrolysed into glucose and enter glycolysis and the TCA cycle, which enhances the energy supply in *P. notoginseng* under heat stress and restores plant growth. Overall, leucine could enhance carbohydrate metabolism by maintaining metabolic homeostasis to alleviate heat stress.

### 4.3 Leucine increases PNSs accumulation by alleviating heat stress

PNSs, which mainly exist in the rhizomes and roots of *P. notoginseng*, exhibit various physiological functions and have different medicinal values (Tang et al., 2022). An increasing number of studies have shown that PNSs need moderate environmental stress and the necessary available resources to accumulate in sufficient quantities to produce medicinal effects (Guo et al., 2020). However, extreme environmental stress can inhibit plant growth and even lead to plant death, thus affecting the accumulation of secondary metabolites (Huang and Guo, 2007). In this study, spraying exogenous leucine at concentrations of 1 and/or 3 mM increased the contents of PNSs in one- and two-year-old *P. notoginseng* under heat stress (**Figures 6E, H**). Considering that the application of leucine alleviates *P. notoginseng* heat stress by enhancing antioxidant capacity and maintaining metabolic homeostasis, we speculated that the application of leucine created a moderate stress environment to allow *P. notoginseng* to resume normal growth and thus increase the contents of PNSs. In addition, PNSs are glycosides formed by the condensation of steroidal or triterpene saponins with sugars or uronic acids, and primary metabolism provides the necessary energy and precursor substances for the biosynthesis of saponins (Liu et al., 2017; Li et al., 2022b). In the saponin synthesis pathway, the initial substrate acetyl-CoA is closely associated with saponin accumulation (Liu et al., 2022). Leucine spraying increased the contents of four organic acids involved in the TCA cycle, so we speculated that the content of acetyl-CoA, as the first metabolite in this cycle, also increased. Moreover, the oxalate content in the stems and roots of *P. notoginseng* increased significantly after leucine application (**Figure 4**). Pan et al. (2015) reported that the addition of oxalate could improve the accumulation of saponins in *Psammosilene tunicoides* cell suspensions, and Li et al. (2022a) reported that the application of oxalate could improve the accumulation of PNSs under Cd stress. It is thus suggested that the increase in oxalate content after leucine application may also contribute to the accumulation of PNSs. The underlying mechanism by which exogenous foliar leucine enhances the accumulation of PNSs deserves further exploration.

## 5 Conclusion

The exogenous application of leucine alleviated heat stress in *P. notoginseng* by improving antioxidant activity and maintaining metabolic homeostasis. The damaged leaf rates of one-year- and two-year-old *P. notoginseng* were significantly decreased after application of 3 and 5 mM leucine both in pots and in the field. Moreover, the dry weight and PNSs content increased after leucine application. These results suggest the potential developing leucine-based plant growth regulators to improve *P. notoginseng* heat resistance.

## Data availability statement

The original contributions presented in the study are included in the article/**Supplementary Material**. Further inquiries can be directed to the corresponding authors.

## Author contributions

SZ and MY conceived and designed the research. HL and YS performed the experiments, collected the plant samples, and analyzed the results. YF, DZ and JX were also involved in the execution of the experiments and sample collection. All authors contributed to the article and approved the submitted version.

## Supplementary material

Supplementary Figure 1 Score plot of OPLS-DA model between nine compared groups. **(A–C)** represent compared groups of L_CK vs L_1, L_CK vs L_3, L_CK vs L_5, respectively. **(D–F)** represent compared groups of S_CK vs S_1, S_CK vs S_3, S_CK vs S_5, respectively. **(G–I)** represent compared groups of R_CK vs R_1, R_CK vs R_3, R_CK vs R_5, respectively.

Supplementary Figure 2 DAMs in leaves, stems and fibrous roots. **(A)** represents the significant up- and down-regulated metabolites treated with 1, 3 and 5 mM leucine in leaves, stems and fibrous roots. **(B–D)** represent venn diagrams of DAMs in leaves, stems and fibrous roots.

Supplementary Figure 3 Metabolic pathways significantly enriched by DAMs. **(A–C)** represent bubble charts of metabolic pathways enriched by DAMs screened from compared groups of L_CK vs L_1, L_CK vs L_3, L_CK vs L_5, respectively. **(D–F)** represent bubble charts of metabolic pathways enriched by DAMs screened from compared groups of S_CK vs S_1, S_CK vs S_3, S_CK vs S_5, respectively. **(G–I)** represent bubble charts of metabolic pathways enriched by DAMs screened from compared groups of R_CK vs R_1, R_CK vs R_3, R_CK vs R_5, respectively. The abscissa represents pathway impact, and the ordinate represents the significance p value after –log10 conversion.

Supplementary Figure 4 Enrichment analysis of DAMs in leaves.

Supplementary Figure 5 Enrichment analysis of DAMs in stems.

Supplementary Figure 6 Enrichment analysis of DAMs in fibrous roots.

Supplementary Figure 7 Venn diagram of metabolic pathway of leaf, stem and fibrous root.

Supplementary Table 1 Explanation rate of cumulative model.

Supplementary Table 2 KEGG enrichment analysis of DAMs.
