# Supplementary figures and images for: Exogenous leucine alleviates heat stress and improves saponin synthesis in Panax notoginseng by improving antioxidant capacity and maintaining metabolic homeostasis
Source: Front Plant Sci. 2023 Apr 19;14:1175878. doi: 10.3389/fpls.2023.1175878 (PMC10154563; doi:10.3389/fpls.2023.1175878)

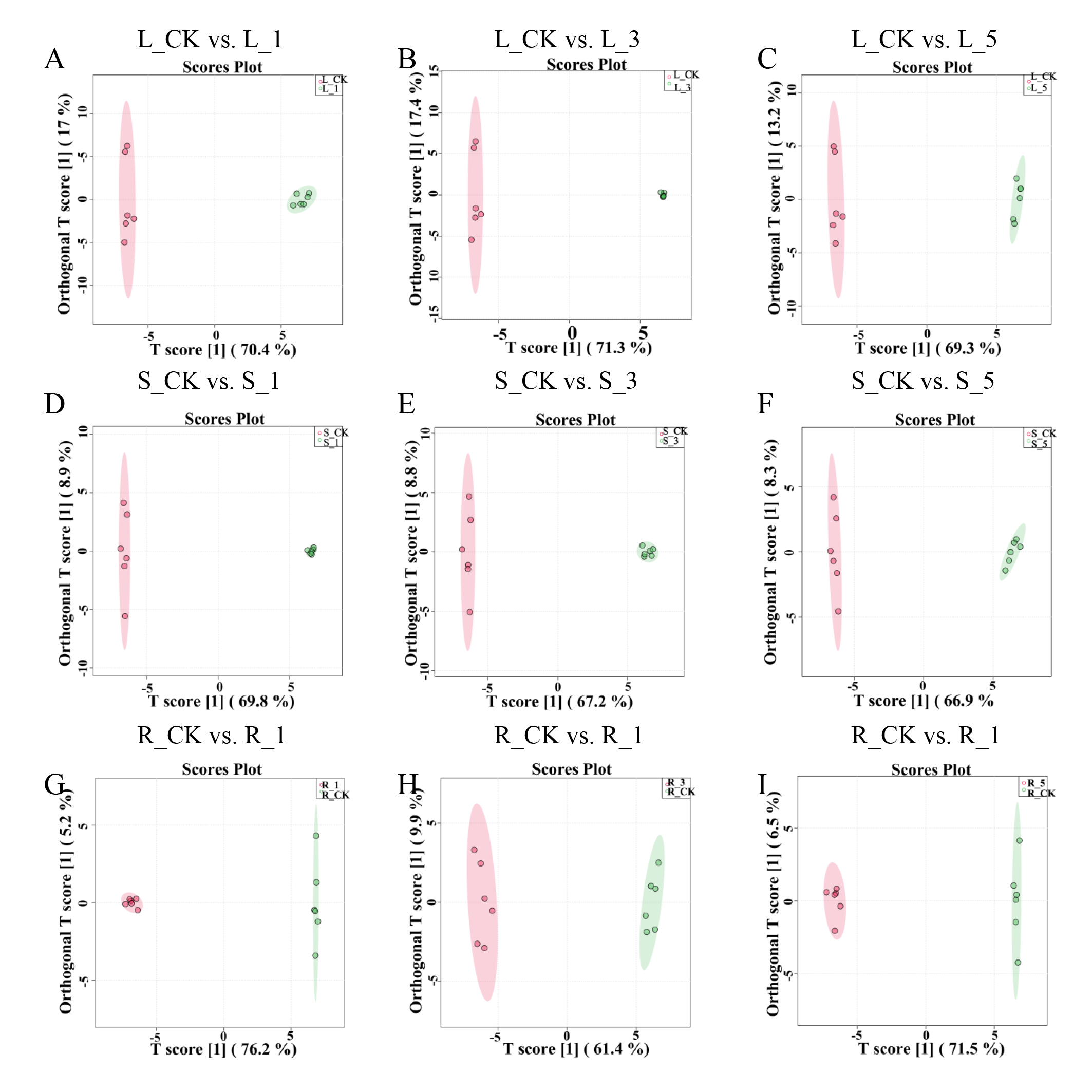

Supplement: Supplementary Figure 1 — Score plot of OPLS-DA model between nine compared groups. (A–C) represent compared groups of L_CK vs L_1, L_CK vs L_3, L_CK vs L_5, respectively. (D–F) represent compared groups of S_CK vs S_1, S_CK vs S_3, S_CK vs S_5, respectively. (G–I) represent compared groups of R_CK vs R_1, R_CK vs R_3, R_CK vs R_5, respectively. [file Image_1.tif]

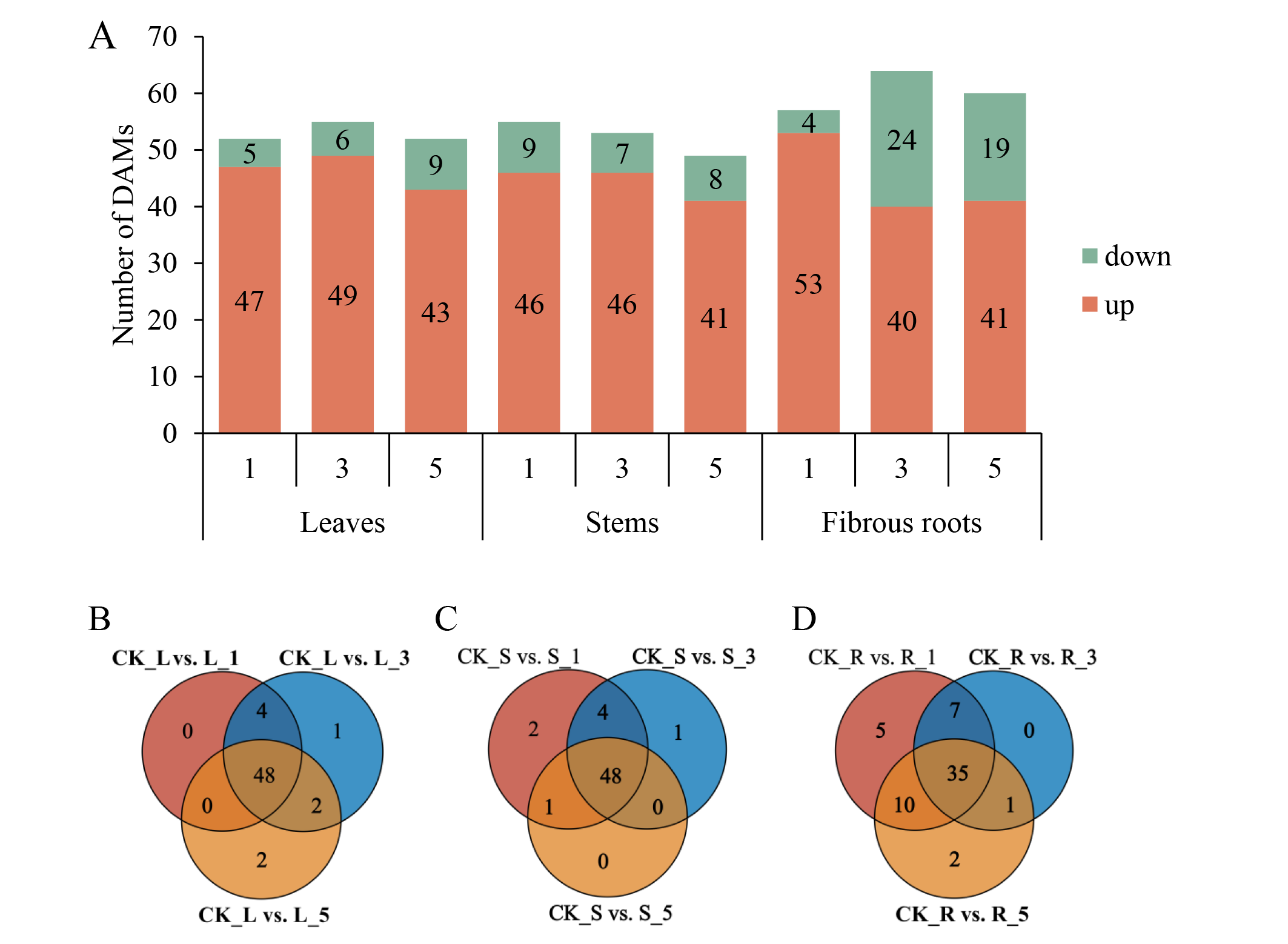

Supplement: Supplementary Figure 2 — DAMs in leaves, stems and fibrous roots. (A) represents the significant up- and down-regulated metabolites treated with 1, 3 and 5 mM leucine in leaves, stems and fibrous roots. (B–D) represent venn diagrams of DAMs in leaves, stems and fibrous roots. [file Image_2.tif]

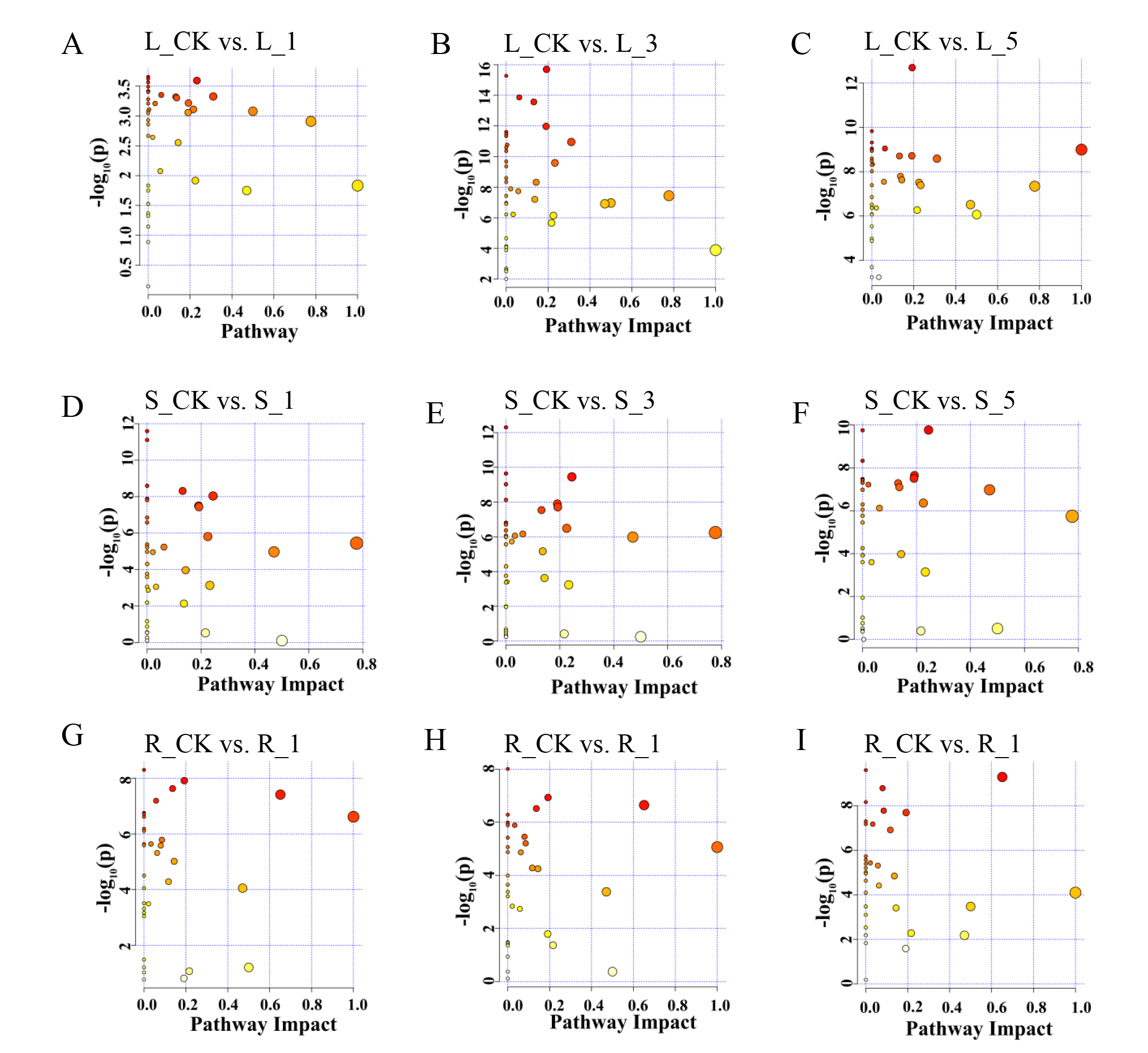

Supplement: Supplementary Figure 3 — Metabolic pathways significantly enriched by DAMs. (A–C) represent bubble charts of metabolic pathways enriched by DAMs screened from compared groups of L_CK vs L_1, L_CK vs L_3, L_CK vs L_5, respectively. (D–F) represent bubble charts of metabolic pathways enriched by DAMs screened from compared groups of S_CK vs S_1, S_CK vs S_3, S_CK vs S_5, respectively. (G–I) represent bubble charts of metabolic pathways enriched by DAMs screened from compared groups of R_CK vs R_1, R_CK vs R_3, R_CK vs R_5, respectively. The abscissa represents pathway impact, and the ordinate represents the significance p value after –log10 conversion. [file Image_3.tif]

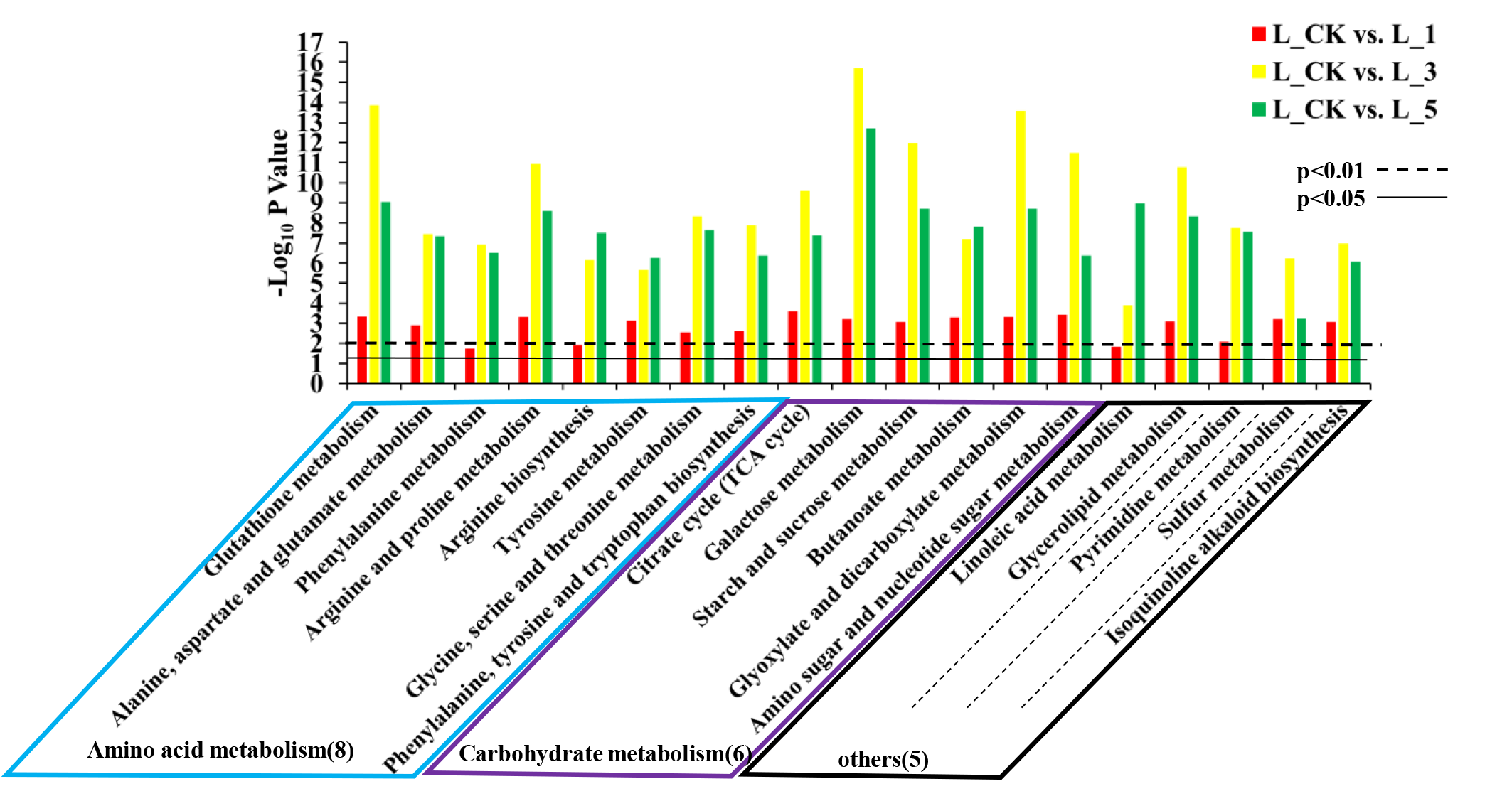

Supplement: Supplementary Figure 4 — Enrichment analysis of DAMs in leaves. [file Image_4.tif]

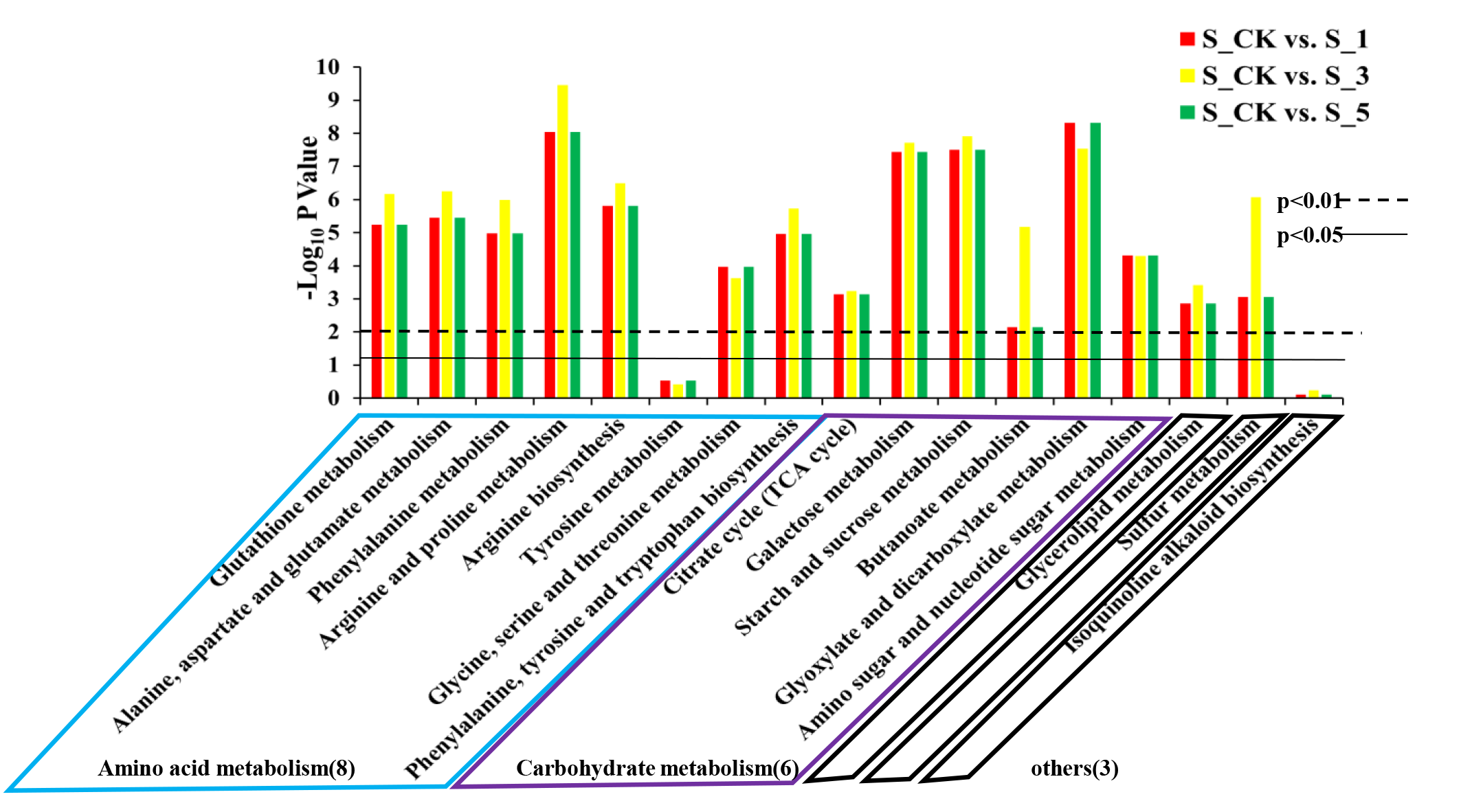

Supplement: Supplementary Figure 5 — Enrichment analysis of DAMs in stems. [file Image_5.tif]

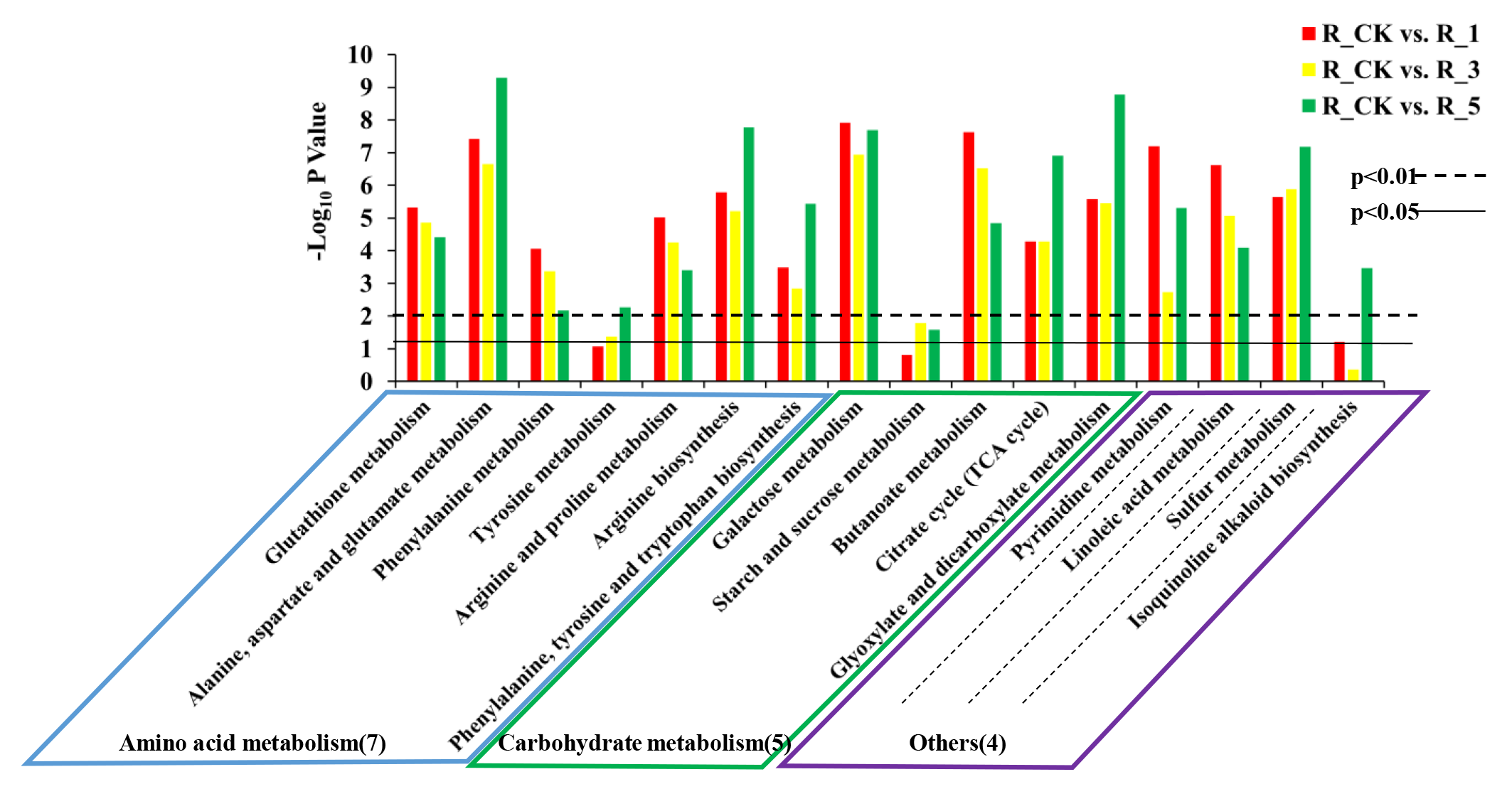

Supplement: Supplementary Figure 6 — Enrichment analysis of DAMs in fibrous roots. [file Image_6.tif]

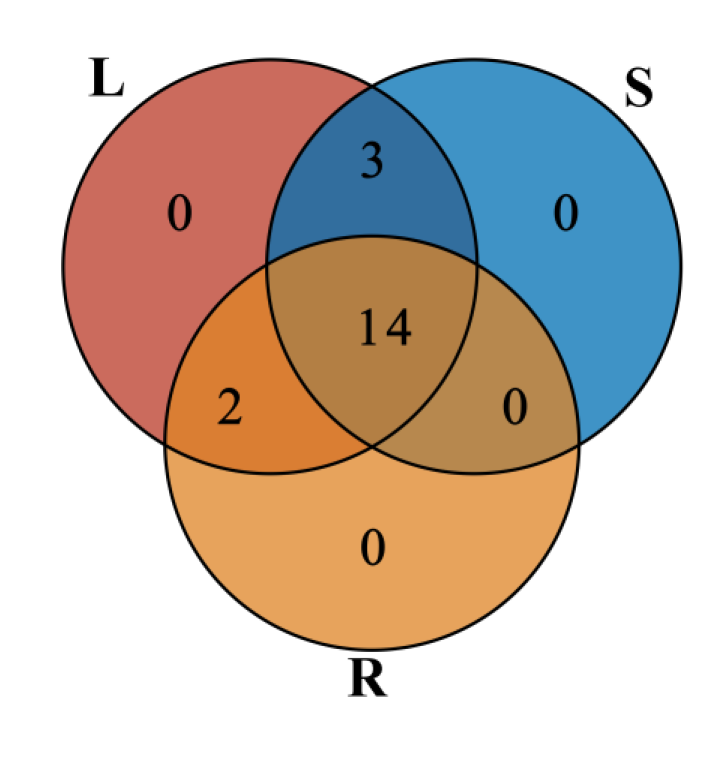

Supplement: Supplementary Figure 7 — Venn diagram of metabolic pathway of leaf, stem and fibrous root. [file Image_7.tif]
